# 1314. Defining Variability in the Evaluation and Management of Children with Chronic Osteomyelitis

**DOI:** 10.1093/ofid/ofac492.1145

**Published:** 2022-12-15

**Authors:** Ganga S Moorthy, Angelique E Boutzoukas, Angelique E Boutzoukas, Daniel Benjamin, Susan E Beekmann, Phillip M Polgreen, John S Bradley, Walter Dehority

**Affiliations:** Duke University Medical Center, Durham, North Carolina; Duke University School of Medicine, Durham, North Carolina; Duke University School of Medicine, Durham, North Carolina; Duke University Medical Center, Durham, North Carolina; University of Iowa, IOWA CITY, Iowa; University of Iowa, IOWA CITY, Iowa; University of San Diego School of Medicine, Rady Children's Hospital, San Deigo, California; The University of New Mexico School of Medicine, Albuquerque, New Mexico

## Abstract

**Background:**

Pediatric chronic osteomyelitis (COM) is an uncommon, poorly defined, debilitating disorder often requiring multiple surgeries and prolonged antibiotic courses. Serious long-term sequelae may occur. As accepted diagnostic criteria do not exist, assessments of disease incidence, medical/surgical approach to management and outcomes are lacking.

**Methods:**

We created a 14-question web-based survey sent to 390 pediatric infectious disease (PID) physician members of the Emerging Infections Network of the Infectious Diseases Society of America. With no standard definition of COM, respondents were asked to conceptualize this disease as they would during routine clinical practice. Of the 148 (38%) survey respondents so far (Table 1), 126 (85%) reported caring for children with COM. We assessed provider characteristics, diagnostic approach, and site-standard surgical and antibiotic management (including oral and intravenous [IV] antimicrobial choice and duration). We performed descriptive statistics on survey results.

**Results:**

Of the 126 respondents, most were >5 years post-fellowship training and nearly half reported managing 3-6 cases of COM per year (Table 2). Prolonged duration of symptoms (79%), normal inflammatory markers (45%), and abnormal plain films (44%) were the most common findings used for diagnosis; however, diagnostic criteria varied. The most common risk factor was orthopedic implants (44%). Management choices and duration are in Figure 1. Most treated with IV antibiotics for < 2 weeks (34%), continuing oral antibiotics for at least 3-6 months (55%). In COM with retained orthopedic implant, most physicians (48%) use oral therapy until implant removal. Considerations for transition from IV to oral antibiotics included improved physical examination (60%), inflammatory markers (52%), extent of disease (55%), causative organism (41%) and location of infection (38%). Though 85% reported being comfortable diagnosing and managing COM, practices varied widely.

**Figure:**
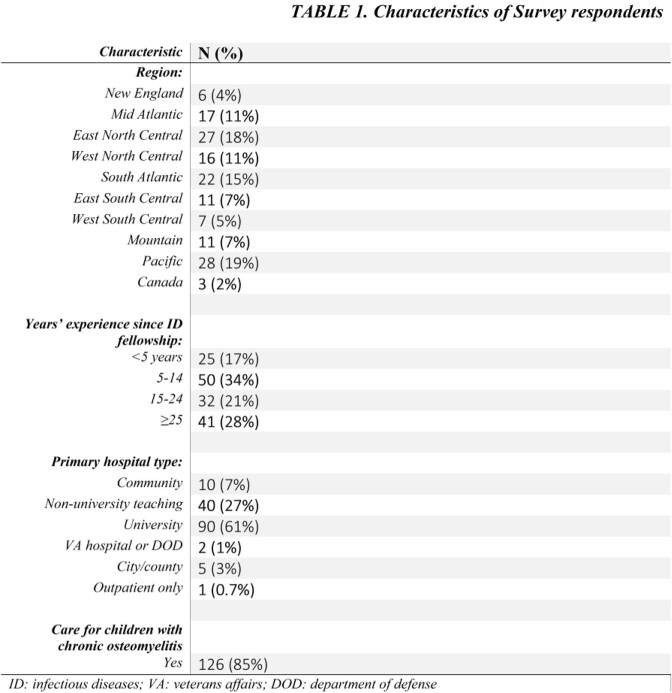

**Figure:**
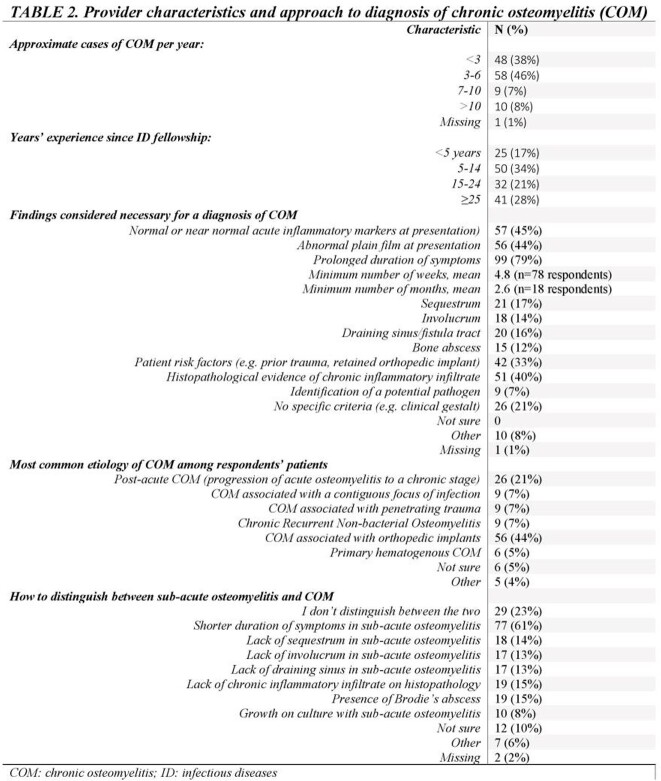

Reported approaches to surgical management (a), duration of IV antibiotics (b), and duration of oral antibiotics (c) in children with COM, and management of COM in patients with implanted hardware (d)

**Figure:**
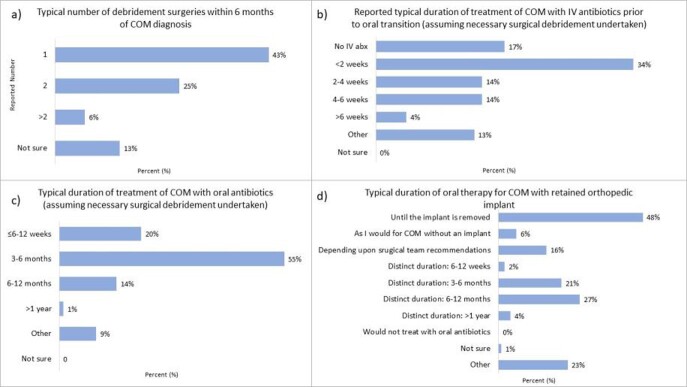

Responses from 126 pediatric infectious diseases physician participating in an Emerging Infections Network survey regarding diagnosis and management of chronic osteomyelitis (COM). Figure 1a) missing data for 2 respondents; figure 1b) missing data for 5 respondents; figure 1c) missing data for 2 respondents; figure 1d) missing data for 9 respondents. COM: Chronic osteomyelitis; IV: intravenous; abx: antibiotics.

**Conclusion:**

Pediatric COM is a complex infection. Formal diagnostic criteria and future prospective studies for medical/surgical management by pathogen/site of infection and presence of foreign material are needed to optimize care and outcomes.

**Disclosures:**

**Daniel Benjamin, Jr., MD PhD MPH**, Allergan: Advisor/Consultant|Melinta Therapuetics: Advisor/Consultant|Syneos Health: Advisor/Consultant.

